# Supplementary material for: Denitrifying bacteria respond to and shape microscale gradients within particulate matrices
Source: Commun Biol. 2021 May 13;4:570. doi: 10.1038/s42003-021-02102-4 (PMC8119678; doi:10.1038/s42003-021-02102-4)
Supplement: Supplementary file 2 — Supplementary Information [file 42003_2021_2102_MOESM2_ESM.pdf]

Supplementary Information for

# Denitrifying bacteria respond to and shape microscale gradients within particulate matrices

Steven Smriga, Davide Ciccarese, and Andrew R. Babbin


I. **Figures**
II. **Methods**
III. **Supplementary Note**
IV. **Supplementary References**

# I. Figures

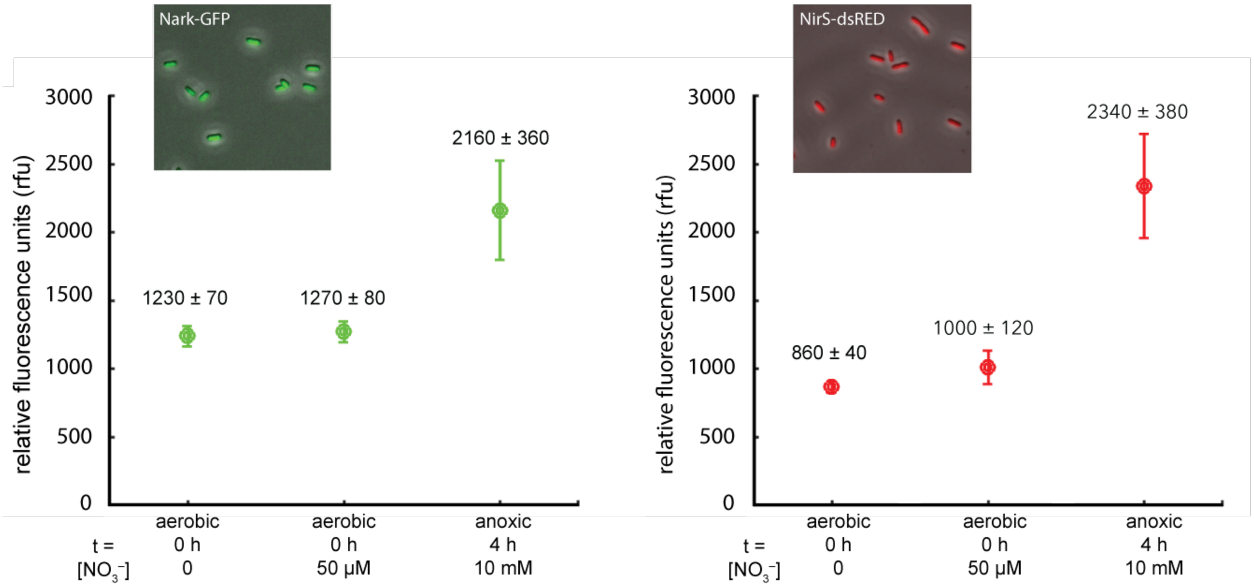


**Supplementary Figure 1. | *Pseudomonas aeruginosa* PAO1 reporter signal validation**. Shown is the relative mean expression of NarK-GFP and NirS-dsRed fluorescence signal from planktonic cells in LB media. Three conditions are shown: cells grown in aerobic conditions overnight (0 h) in the absence of nitrate, cells grown in aerobic conditions overnight in the presence of nitrate (50 µM), and cells 4 h after transfer from aerobic conditions to anoxic conditions in the presence of nitrate (10 mM). The mean signal increase in anoxic conditions indicates that each reporter strain reflects upregulation of nitrate reductase (NarK-GFP) or nitrite reductase (NirS-dsRed), respectively. All treatments for both NarK-GFP and NirS-dsRed are significantly different from each other (NarK-GFP, One way ANOVA, $F_{1,4071} = 8.6 \times 10^3$, $p < 0.001$; NirS-dsRed, One way ANOVA, $F_{1,6660} = 2.7 \times 10^4$, $p < 0.001$).

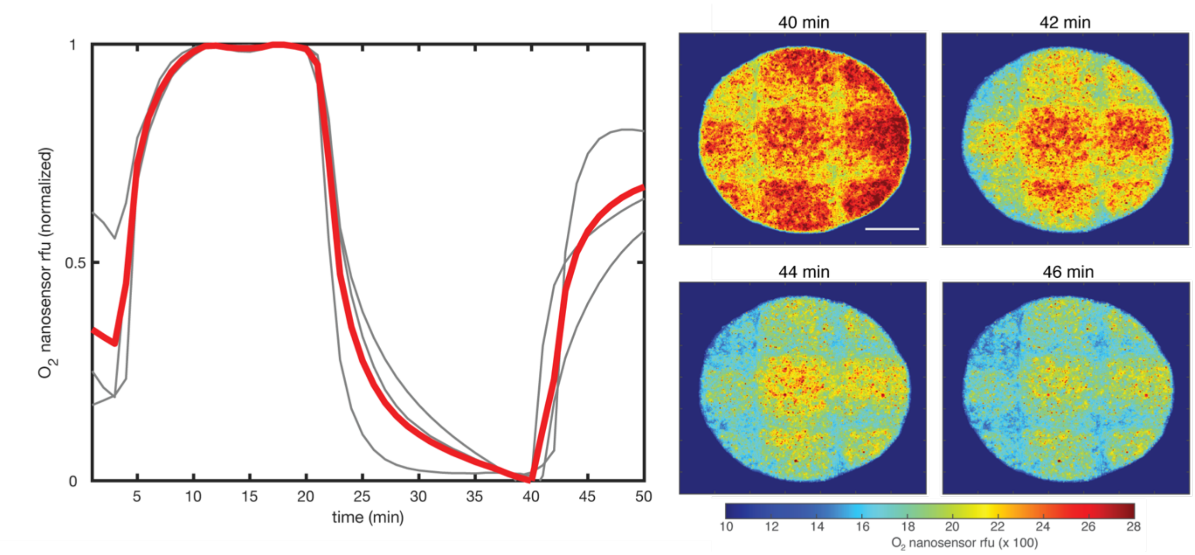


**Supplementary Figure 2. | Validation of oxygen nanosensors to report oxygen dynamics within model agarose particles in gastight devices**. Shown is the mean fluorescence signal (red thick line) for oxygen nanosensors of three replicates during rapid switching between air- and $N_2$-saturated PBS for three replicates particle in the absence of bacteria. Higher oxygen nanosensor fluorescence corresponds with lower oxygen concentration. Upon switching from $N_2$ to air, mean particle oxygen nanosensor signal decayed by ~90% over 6 min. Air diffused laterally from the particle edge inward to the particle core.

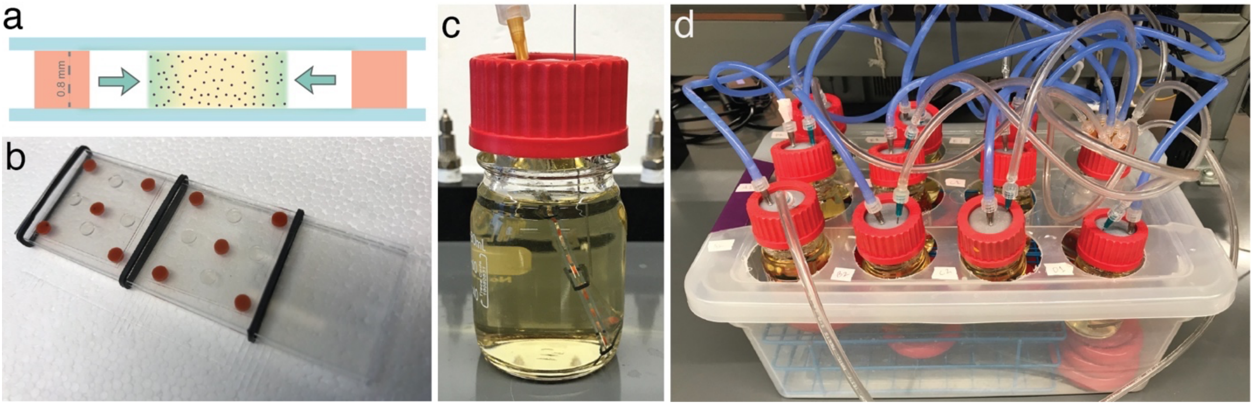


**Supplementary Figure 3. | The 'medusa' system enables stable gas conditions in fluid surrounding particles**. **a,** Diagram illustrating a model particle embedded with bacteria and oxygen nanosensors held between glass coverslips separated by silicone discs. Glass surfaces prevent vertical diffusion while permitting visualization upon transfer to the microscope. Nutrients and gas in bulk fluid can diffuse laterally into the particle. **b,** A 'domino' mount with two coverslip mounts each holding four particles held between glass slides bound by small rubber bands. **c,** A domino mount submerged in a gastight glass bottle with a lid punctured by a sparging gas syringe needle and an exhaust syringe needle. **d,** Ten gastight bottles each with an incoming sparging gas line and an outgoing exhaust line, with the latter connected to a catchment bottle to prevent aerosolization into the laboratory. Bottles are maintained in a water bath for temperature stability.

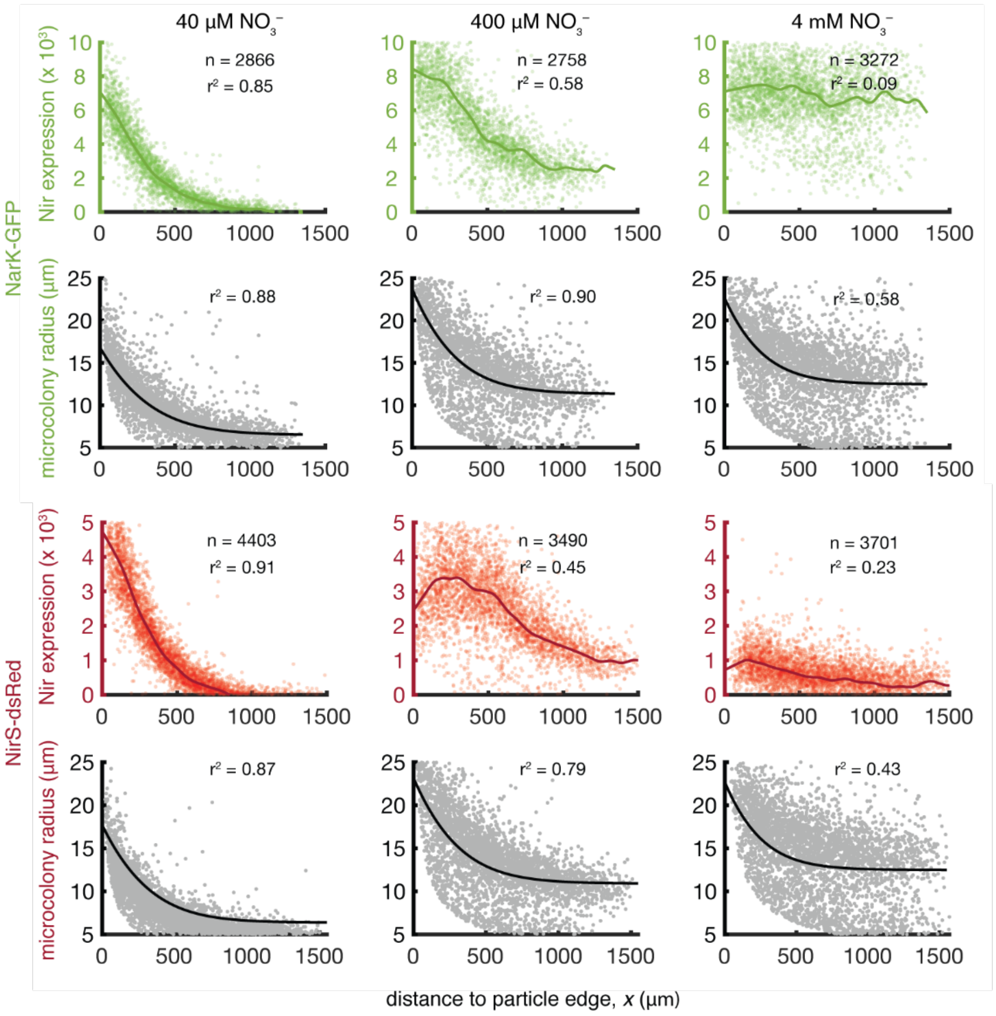


**Supplementary Figure 4. | Denitrification gene expression and microcolony size within particles surrounded by fully anoxic fluid.** Results for a single replicate showing PAO1 microcolonies expressing NarK-GFP (nitrate reductase) or NirS-dsRed (nitrite reductase) after 40 h of growth in bulk anoxic media supplemented with 3 nitrate concentrations. The number of microcolonies *n* for each replicate is indicated. The mean is indicated for Nar or Nir expression (green or red curve, respectively), and an exponential model fit for radii is indicated (black curve), as a function of radial distance from the particle edge. Mean results for multiple replicates are shown in the main text (Fig. 2).

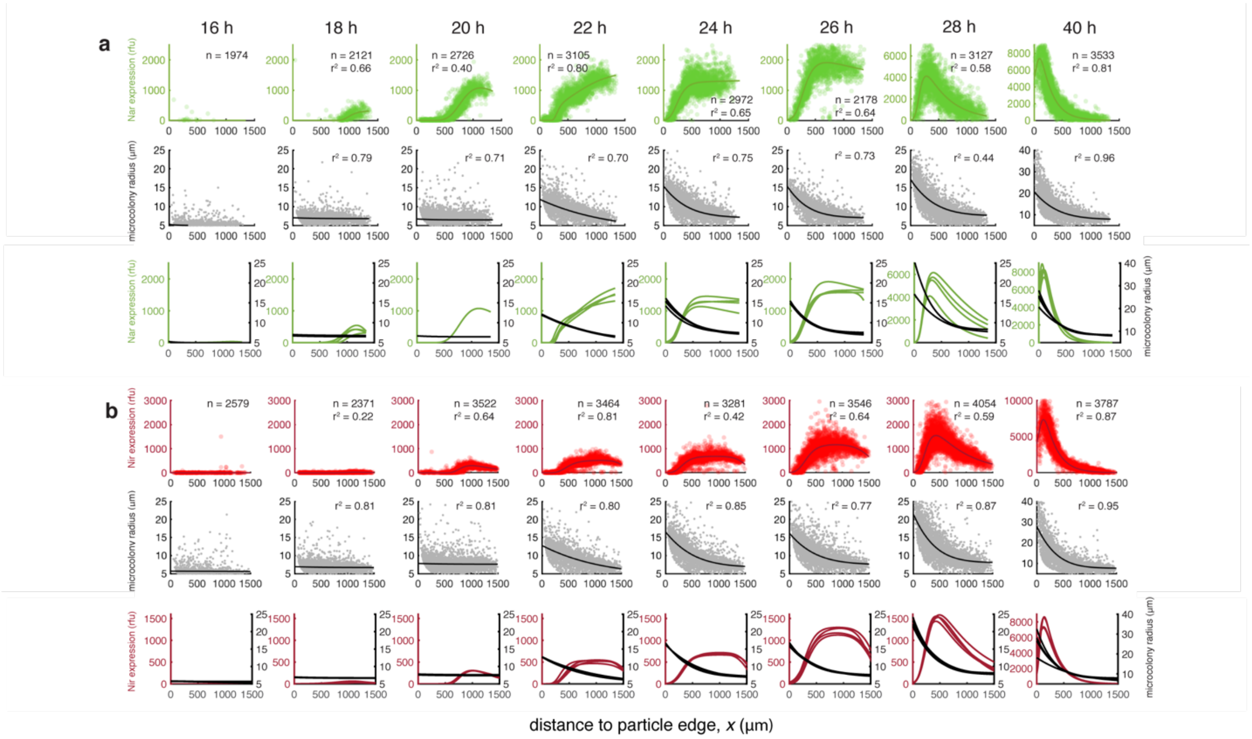


**Supplementary Figure 5. | Onset and radial migration of PAO1 denitrification expression in particles, coupled with microcolony growth. a,** Results for NarK-GFP and **b**, results for NirS-dsRed, showing microcolony attributes as a function of radial distance from the particle edge during a time-course. Columns delineate timepoints. For each, the top row shows mean expression for *n* microcolonies in one particle overlayed with a customized curve fit and the goodness of fit $r^2$ (see Supplementary Note); the middle row shows the radius *r* for the same microcolonies with an exponential curve fit and the goodness of fit $r^2$; and the bottom row shows the curve fits for all replicates (ranging from 2 to 4) at each timepoint. For all, bulk fluid was maintained at 50% air saturation.

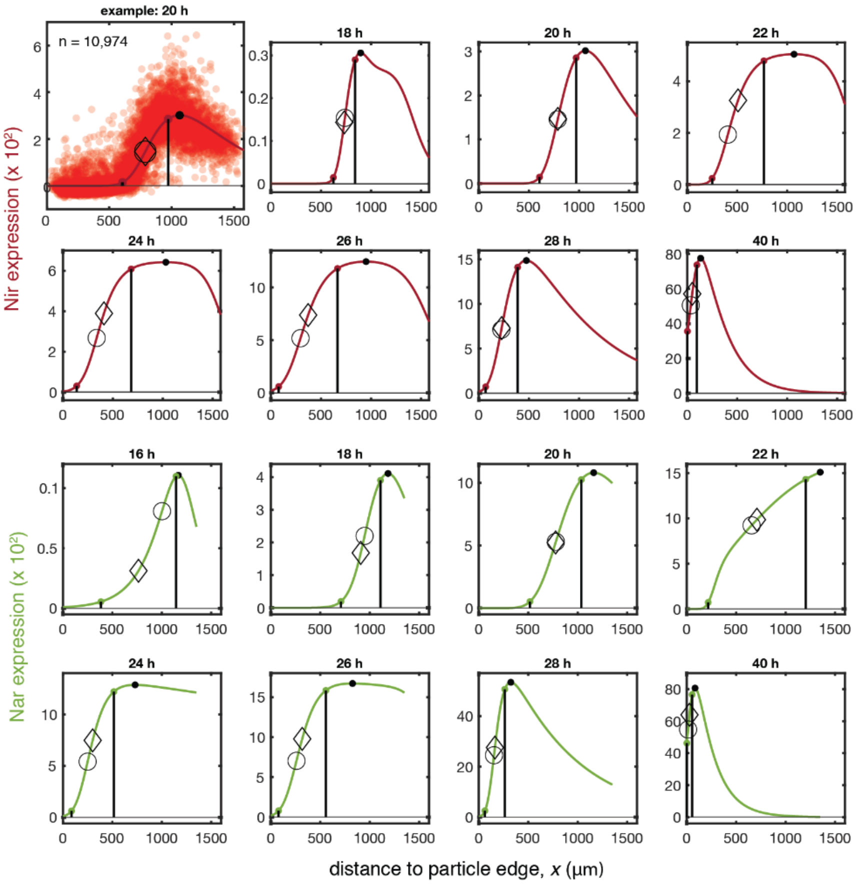


**Supplementary Figure 6. | A migrating wave of denitrification gene expression is indicated by a transition zone.** For NirS-dsRed (top) and NarK-GFP (bottom), these plots illustrate the radial location and widths of a transition zone for each timepoint, approximated as the sloped region closest to the particle edge in the expression profile. An illustration for a single timepoint (upper left, NirS-dsRed, $t = 20$ h) shows the microcolony data used to create the mean curve fit of up to four replicates. The maximum value of each curve fit is indicated by a black filled circle. The transition zone outer boundary (0.05 of the max value) and the inner boundary (0.95 of the max value) are indicated by red filled circles and vertical black lines; the width between these two points is shown in the main text (Fig. 3e). The midpoint of the slope (open diamonds) and the inflection point of the slope (open circles) are also indicated (also in Fig. 3e). There was no transition zone for $t = 16$ h for NirS-dsRed (*i.e.*, no expression detected).

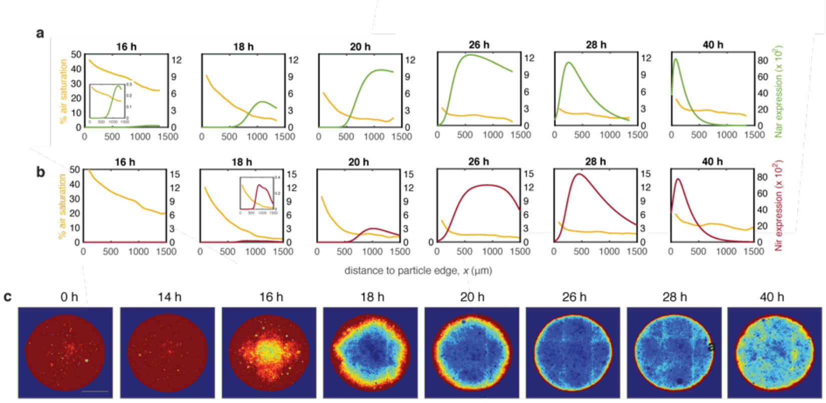

**Supplementary Figure 7. | Evolution of anoxia within particles surrounded by partially aerated fluid.** **a,** Air saturation profiles compared with Nar expression and **b,** compared with Nir expression, for a subset of timepoints. Profiles of air saturation were generated from particles co-seeded with PAO1 reporter strains and oxygen nanosensors. For Nar or Nir expression, the normalized mean curve fit from up to four replicates particles for each timepoint is shown; Nir expression at $t$ = 16 h was negligible. Insets highlight low but detectable expression in early time points that is obscured at larger scale. **c,** Two-dimensional air distribution in particles co-seeded with the NirS-dsRed reporter strain (nitrite reductase) and oxygen nanosensors. Scale bar = 700 µm. Air distributions were similar to those created by NarK-GFP (Fig. 4).

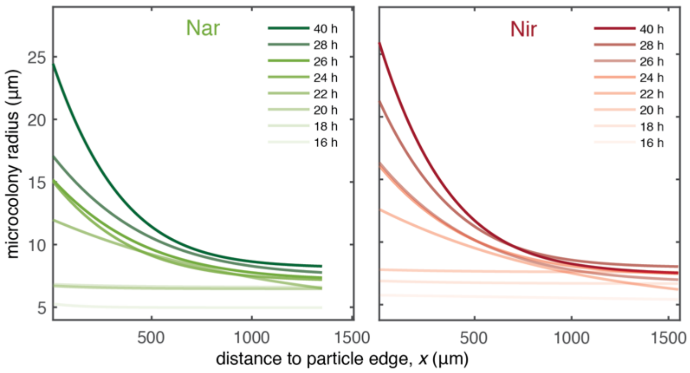


**Supplementary Figure 8. | Microcolony radii for Nar and Nir microcolonies.** Microcolony size was quantified throughout the period of radial migration of denitrification expression within particles in partially aerated fluid, which corresponds with results shown in the main text (Fig. 3) and Supplementary Fig. 5. A mean exponential model fit of microcolony radii for each particle is shown for each reporter strain for each timepoint during the migration.

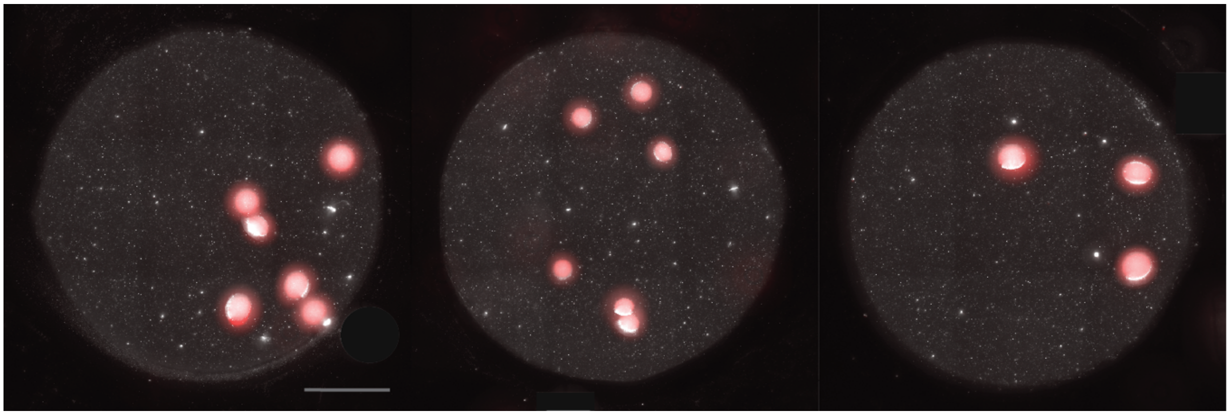

**Supplementary Figure 9. | Low seeding density in particles yields large microcolonies.** Shown are maximum projection images for three of nine replicate particles with PAO1 NirS-dsRed. Scale bar = 750 µm. In contrast to other experiments (see Figs. 1–5), particles were seeded at very low density (~$5 \times 10^2$ cells $mL^{-1}$), resulting in 1 to 6 microcolonies $particle^{-1}$. After 40 h of growth, the mean microcolony radius *r* was 61.7 ± 11.1 µm (sd), which remained approximately the same after 66 h total (shown in these images). The mean radius (*r* ~ 61.7 µm) is >2.3-fold larger than the mean of the microcolony radii that grew at a density of ~7000 microcolonies per particle under similar conditions (50% air, 40 µM $NO_3^-$) for an equivalent duration (Supplementary Fig. 7).

## II. Methods

**Model denitrifier organism.** Our study used two fluorescent reporter strains of *Pseudomonas aeruginosa* PAO1. One has the promoter region of nitrate reductase (*nar*$K_1K_2$GHIJ) fused to eGFP (herein termed NarK-GFP) and the second has the promoter region of nitrite reductase (*nir*SMCFDLGHJEN) fused to DsRed-Express (herein termed NirS-dsRed)[1,2]. Strains were provided courtesy of Masanori Toyofuku and Nobuhiko Nomura (University of Tsukuba, Japan). The promoter fusion used in this study does not influence the down-stream expression of the reductase genes.

**Reporter strain signal validation.** Planktonic cells of PAO1 NarK-GFP or NirS-dsRed were inoculated into 20 mL of Luria-Bertani Broth (BD Difco™ LB Broth (Miller), product number 244620) supplemented with gentamycin (Gm; 30 µg/mL) in a 125-mL Erlenmeyer flask. Cultures were incubated at 37 °C overnight, diluted 1:200 into 20 mL of fresh media and again incubated at 37 °C for ~1 h with shaking at 120 rpm. This assured that cultures were fully aerated and undergoing aerobic respiration. Following a previous study[2], culture aliquots were then transferred into 15 mL of $N_2$-sparged anoxic LB media in sealed serum bottles and incubated 4 h with shaking at 120 rpm. Oxic fluorescence recovery (OFR) was applied by opening the serum bottle, adding chloramphenicol (50 ng $mL^{-1}$) to cease protein synthesis, incubating 1 h aerobically with shaking, and then adding formaldehyde to fix cells for 30 min. Fixed cells were then washed 2× in phosphate buffered saline (PBS). For imaging, a 15 µL aliquot was mounted to a glass slide beneath a coverslip (22 × 22 mm), which was then pressed downward gently to dispense fluid. The prep was incubated 20 min prior to imaging which allowed floating cells to settle and adhere to glass surfaces. Notably, in the absence of OFR, fluorescence signal was indistinguishable from background for live cells of both PAO1 NarK-GFP and NirS-dsRed, owing to a lack of oxygen required for maturation of eGFP and dsRed fluorescence. OFR followed by fixation greatly increased the fluorescence signal to background[2,3].

**Fabrication of model particles seeded with PAO1 and/or oxygen nanosensors.** Model particles were composed of 1% low melt agarose (Sigma-Aldrich, #A4018) seeded with PAO1 reporter cells. To prepare them, agarose (0.1 g) was mixed into filter-sterilized PBS (11 mL), covered, and heated to dissolve. The cover was removed and heating continued ~10 min until the volume was reduced to 10 mL. Molten agarose was removed from the hot plate and cooled to 37–40 °C. In parallel, PAO1 cells (NarK-GFP or NirS-dsRed) were grown aerobically (LB media + $Gm_{30}$, ~16 h, 37 °C, 180 rpm), then diluted into fresh media (same growth conditions) ~3 h prior to agarose casting. This ensured cells were in aerobic log phase growth and using oxidative respiration. The aerobically-grown cells were diluted in LB media to a target $OD_{600}$ ~ 0.15. When the molten agarose had cooled (~40 °C), cells were mixed into it at various volumes, *e.g.*, for a cell

diluent with $OD_{600}$ = 0.16, the addition of 133 µL of cells to 10 mL of molten agarose achieved a seeding density of ~$10^6$ cells $mL^{-1}$. For a subset of particles, oxygen nanosensors were embedded by mixing them into the cell-agarose mixture to create a final concentration of 50 µg $mL^{-1}$ (or 5 mg $mL^{-1}$ in preliminary experiments, Supplementary Fig. 2).

The cell-amended or cell + oxygen nanosensor-amended molten agarose was drawn into a pre-warmed syringe, a blunt end needle was attached (22G; Industrial Dispensing Supplies), a small volume was dispersed to exude bubbles, and then ~1.5 mL was injected to a gel casting setup. The casting setup comprised two glass slides (1 in × 3 in) separated by thin strips of clear silicone sheet (McMaster-Carr #87315K63, 0.020 in thickness) stacked atop each other (*i.e.*, to create 0.04 in thick separators) arranged on the two long edges of the slides. After the cast gels had solidified, one side of the casting glass was removed. Discs of 3 mm diameter were punched into the agarose using a sterile tissue biopsy punch (Sklar Instruments, Tru-Punch, #96-1144), then a sterile razor blade and forceps were used to remove the discs and transfer them into a millifluidic device or a 'domino' coverslip mount, dependent on experiment (see below).

**Gastight, continuous-flow millifluidic device.** Based on a previous design[4], we fabricated a millifluidic device for tight gas control in microenvironments (Fig. 1). In contrast to commonly used PDMS-based devices, our device is constructed with three gas-impermeable glass slides (the bottom is a 2 in × 3 in slide, the top consists of two 1 in × 3 in slides) that permit model agarose particles to be held within a channel. Glass slides were held separated by red silicone sheet (Diversified Silicone Products, #5038GP-032, 50 Duro, 1/32 in thick; McMaster-Carr #1460N21) trimmed to create two channels on each device. The model agarose particles, *i.e.*, agarose discs, were sandwiched so that media could penetrate only laterally from the particle's margins, allowing the central region to become completely anoxic under bacterial respiration (Fig. 1a). The millifluidic channels enabled one-directional fluid flow around the stationary agarose particles such that bulk conditions were held constant. LB media was saturated with varying concentrations of $O_2$ (balanced with $N_2$ and $CO_2$) using mass flow controllers (Alicat Scientific); the media was held in 2 L glass bottles and was pre-equilibrated at a high gas flow rate (~300 mL $min^{-1}$) then subsequently reduced so that the experimental gas flow was much lower (~0.275 mL $min^{-1}$). All milli-fluidic experiments were conducted at room temperature (21±1 °C). Thus, flow in the system was driven by gas pressure. Tubing with low oxygen permeability (Viton fluoroelastomer rubber, 1/16 in inner diameter, McMaster-Carr #5119K39) connected the 2 L media source bottles to the microscope-mounted millifluidic device. The connector between the tubing and the device was a 0.083 in outer diameter stainless steel 90-degree angled connector with 1 in legs (McMaster-Carr #5560K85).

Flow is critical to advective solute transport; in the absence of flow, the diffusive flux of $O_2$ into particles should decrease, as well as the transport of nitrogen intermediates (*e.g.*, $N_2O$) away from the particle[5]. While silicone sheeting comprising the device's channel walls is gas permeable, high velocity fluid flow (~0.7 mm $s^{-1}$; channel residence time ~ 56 s) in these experiments sustained laminar flow and as such, any fluid oxygenated adjacent to the silicone walls did not entrain into fluid that encountered agarose particles. A major advantage of this gas pressure-driven millifluidic system, relative to a vertical flow-through chamber used to suspend organic aggregates (*e.g.*, ref. 6), is the ability to microscopically quantify the dynamics and activity of bacteria within the suboxic/anoxic particle microhabitat.

**Validation of oxygen nanosensors to report oxygen dynamics.** A subset of particles were embedded with oxygen nanosensors[6] which can be visualized via fluorescence microscopy to directly quantify oxygen concentrations in two dimensions.

We first confirmed that oxygen nanosensors could be cast within agarose and that fluorescent signal could be clearly differentiated between air- and $N_2$-saturated PBS within a gastight millifluidic device (Supplementary Fig. 2). To do this, agarose particles embedded with oxygen nanosensors, but without PAO1 cells, were mounted into two channels of the millifluidic device. The pressure-driven bulk fluid system (see above) was filled with PBS; one bottle was air-saturated and attached to one millifluidic channel, the other bottle was $N_2$-saturated and attached to the second millifluidic channel. Flow was induced at ~0.275 mL $min^{-1}$ to create a flow velocity of ~0.7 mm $s^{-1}$ around particles. Images were captured continuously every 2 min. After several minutes, the fluid source to each channel was switched, *i.e.*, the flow was stopped, pinch clamps were applied to Viton tubing, the tubing was switched, the clamps removed, and the flow was reinitiated. The signal was monitored visually and, when steady state was reached after ~30 min in each particle, the fluid sources were switched back. Again, steady state was achieved after ~30 min of flow (not shown), and the experiment was terminated.

We then tested oxygen nanosensors in agarose particles co-embedded with PAO1 at a density of $10^6$ cells $mL^{-1}$. Particles were mounted in the millifluidic device, then air-saturated LB media amended with $NO_3^-$ (final concentration ~40 µM) flowed around the particles over ~24 h, while images for oxygen nanosensor fluorescence were captured each 30 min. After a delay of ~14 h accounting for bacterial growth, oxygen nanosensor signal increased in the particle indicating the development of suboxia even within the air-saturated bulk flow field. The suboxia occurred first in the center core and then extended outward to the periphery over ~7 h (results not shown). A subsequent experiment repeated this except using particles with three seeding densities ($10^4$ – $10^6$ cells $mL^{-1}$), and the results are shown in Fig. 1d-e.

These initial experiments used agarose particles embedded with oxygen nanosensors at 5 mg $mL^{-1}$ and images were captured with a fluorescent filter set not optimized for the oxygen nanosensor emission

spectrum (CY5, Chroma 49006). Subsequent biological experiments used oxygen nanosensors embedded at 100-fold lower concentration (0.05 mg $mL^{-1}$) and a more optimal fluorescent filter set (see below). With either filter set, higher oxygen nanosensor signal corresponds with lower oxygen concentration.

We converted oxygen nanosensor fluorescence signal to air saturation using the Stern-Volmer equation $I_0 / I = 1 + K_q[O_2]$. The value $I_0$ was assumed to be the fluorescence intensity in the absence of oxygen, *i.e.*, the brightest intensity in the particle core. $K_q$ is a quenching constant which was determined by rearranging the equation for each experimental condition, *e.g.,* when bulk fluid was set to 50% air saturation ($I_{50}$) then $K_q = (I_0 / I_{50} - 1) / 50$. Then, for any measured pixel intensity $I$ we calculated the associated oxygen content as *% air saturation* = $(I_0 / I - 1) / K_q$. Though it would have been ideal to calibrate with $N_2$-saturated agarose, this would have been required for each specific hydrogel preparation, because each had a slightly variable oxygen nanosensor concentration and associated mean background fluorescence signal. In other words, the condition $I_0$ would have a different mean fluorescence signal for each hydrogel preparation, and since PAO1 cells were co-seeded into each hydrogel, inducing full anoxia with $N_2$-saturated fluid would have disrupted the denitrification response we aimed to quantify.

**Time-course for quantifying the evolution of anoxia and PAO1 denitrification expression in model particles using the 'medusa' system.** Agarose particles of ~3 mm diameter were embedded with either NarK-GFP or NirS-dsRed at $10^6$ cells $mL^{-1}$, equivalent to ~7000 cells per particle. Because of the requirement to expose live reporter cells to oxygen in order to quantify fluorescence over many replicate particles, we developed a system for sacrificial endpoint sampling, herein termed the 'medusa' system (Supplementary Fig. 3). Particles were transferred to glass 'domino' coverslip mounts. Each mount contained a 22 × 22 mm coverslip to which 3 mm circular separators of silicone sheeting (Diversified Silicone Products, #DSP5038GP-032, 50 Duro, 1/32 in thick; McMaster-Carr #1460N21) had been bonded. After 4 agarose discs were transferred, a second coverslip (with no bonded separators) was placed atop it, then this stack was placed between two glass slides (1 in × 3 in) which were then bound together with 3 - 4 rubber bands (Supplementary Fig. 3). Four Nar-GFP and four Nir-dsRed particles, each within their own domino coverslip mount, were held within one pair of glass slides. Like the millifluidic device, the domino mounts permitted only lateral diffusion of nutrients into particles.

The particles in the domino mounts were then submerged in a set of gastight glass bottles with silicone septa that could be punctured with needles (Pyrex Media Bottle Cap, GL45, #1395-45HTSC; Pyrex Septa, GL45, PTFE Faced Silicone, #1395-45TS). The bottles contained 100 mL of LB media amended with gentamycin (30 µg/mL), antifoam B silicone emulsion (10 µL; J.T. Baker, #B531-05), and 40 µM $KNO_3$. LB media was saturated with 50% air (balanced with $N_2$ and ~1% $CO_2$) that bubbled continuously at a flow rate of 100 mL $min^{-1}$ through a 22G × 4" needle (Air-Tite Products Co., Inc.). The gas headspace of each

bottle was vented through a syringe needle outlet connected to tubing that connected to a separate bottle trap to prevent aerosolization of PAO1 (Supplementary Fig. 3).

The results of a preliminary experiment demonstrated microcolonies were very small through ~12 h of incubation. In the subsequent experiment (Fig. 3), following an initial ~14 h of incubation, particles were sampled sacrificially every ~2 h, which included imaging of the oxygen profile using one replicate particle that had been co-embedded with oxygen nanosensors (50 µg $mL^{-1}$) for both NarK-GFP and NirS-dsRed. At each sampling point, domino mounts were removed from incubation bottles and the oxygen nanosensor-embedded particles were imaged within 5 min. To do this, the domino mount and the suboxic LB media from the same bottle were transferred to a one-well plate (127.8 × 85.5 mm; Thermo Scientific Nunc, #267060) and the mount was held stable in the plate with neodymium magnetic discs (McMaster-Carr, #5862K141). On the microscope, an image was made for two oxygen nanosensor-embedded particles, one co-seeded with NarK-GFP, the other with NirS-dsRed. The domino mounts were then disassembled, the particles transferred to a 48-well assay plate, and oxic fluorescence recovery (OFR) was applied (see previous). Like planktonic cells, fluorescence signal was negligible for live cells in agarose discs, but it was greatly improved through application of OFR, fixation, and washing of the discs after incubation. Following a 3× wash in PBS, particles were suspended in PBS containing 1% w/v of the antioxidant Trolox (6-hydroxy-2,5,7,8-tetramethyl-chromano-2-carboxylic acid; Sigma-Aldrich, #238813). The well plate was covered with aluminum foil sealant and stored at 4 °C for 1–3 days prior to imaging of microcolonies. Separate fluorescence stability experiments confirmed this period did not affect the fluorescence signal.

Subsequently, an experiment was performed to evaluate fluorescent signal within individual microcolonies (Fig. 5). The time-course evolution experiment described above was repeated (LB media with 40 µM $NO_3^-$, saturated with 50% air) but particles were sacrificially sampled for only one timepoint ($t = 28$ h). Higher resolution images were attained around the periphery of two replicate particles embedded with NarK-GFP and two replicates embedded with NirS-dsRed.

**Time-course for quantifying PAO1 denitrification expression over a range of bulk nitrate concentrations.** The design and details of this experiment were the same as the time-course for quantifying the evolution of anoxia except for the following aspects. First, the LB media contained different concentrations of nitrate: 40 µM, 400 µM, or 4 mM. Second, the bottles were held in stable anoxic conditions, *i.e.*, they were saturated via bubbling with 99.8% $N_2$ balanced with 0.2% $CO_2$. Third, given the prescribed anoxia, no particle embedded with oxygen nanosensors was prepared nor imaging conducted for oxygen profiles. Lastly, particles were sacrificially sampled at just one timepoint ($t = 40$ h).

**Low-seeding density.** A separate experiment was performed to microscopically evaluate microcolony growth in particles at low seeding density in the gastight millifluidic device (Supplementary Fig. 9). Nine replicate particles with PAO1 NirS-dsRed were seeded at low density (~$5 \times 10^2$ cells $mL^{-1}$) resulting in 1 to 6 microcolonies per particle. The experimental conditions described above were repeated (LB media with 40 µM $KNO_3$) but saturated with 100% air. Images were acquired every 30 min for 66 h, after which particles were fixed and then a subset of replicates were imaged.

**Statistics and Reproducibility**. All statistical tests were conducted in Matlab (R2019a) using parametric tests in the case of normally distributed data; otherwise non-parametric tests were used. The ANOVA tests were conducted for normally distributed data considering $p$-values < 0.05 to be statistically significant, although the resulting $p$-values were generally < 0.001. Denitrification gene intensity was fit using the Matlab curve fitting toolbox (Supplemental) and colony sizes using Matlab nonlinear regression assuming an exponential of the form, $Size = k_1 e^{-k_2 r}$.

**Microscopy and image analysis.** All imaging was performed on a Nikon Ti2-E inverted microscope fitted with an Andor Zyla 4.2 sCMOS camera and a SOLA SEii 365 Light Engine controlled via Nikon Elements (v.4). Images were captured in 16-bit with a 10× (Plan Fluor DLL) or 40× objective lens (Super Plan Fluor ELWD), the latter to quantify NarK-GFP and NirS-dsRed signal at the scale of individual microcolonies. The following fluorescent filter sets were used: EGFP (Chroma 49002, ex 470/40 nm, em 525/50 nm) for NirK-eGFP signal, CY3/TRITC (Chroma 49004, ex 545/25 nm, em 605/70 nm) for NirS-dsRed signal, and a customized set herein termed 'CY5-LP' (ex 620/60 nm, em >665 nm, Chroma ET665lp) for oxygen nanosensor signal in the near-infrared. For microcolony expression and size, we captured images with a 20 µm z-step over ~650 µm depth. Image analyses were performed within Nikon Elements and Matlab (R2019a) via customized scripts.

For expression within individual microcolonies (Fig. 5), images were captured with a 5 µm z-step over ~110 µm depth. Each brightfield image was adjusted via contrast-limited adaptive histogram equalization followed by a Gaussian filter (smoothing kernel, sigma= 5 px). Each colony was then identified using a Sobel edge detection algorithm. The Sobel algorithm was set using a factor of 1.15 multiplied by the threshold previously calculated using the Sobel algorithm itself. The image was then processed via a connected component analysis in order to fill interior gaps and then segment colonies.

To quantify the area of the colony expressing the fluorescent reporter of the target gene (NarK-GFP or NirS-dsRed), we built a local threshold as follows. For each microcolony, the fluorescence background signal was sampled in the adjacent surrounding area with a defined radius (25 px), and the local maximum intensity value of each colony was stored. These local maximum intensities sampled near

colonies across the whole image were averaged, and this was used as a threshold to binarize the fluorescence reporter signal within each microcolony. A binary image of the segmented brightfield image was used to identify the region of interest in the fluorescence channel, and the two-dimensional total area of each colony was quantified. A fraction of the fluorescent reporter 'on' was calculated by dividing the binarized fluorescence area against the two-dimensional total area of each colony. The fluorescence colony fraction 'on' was then plotted with bins of 150 μm radial particle space, as well as a probability density function to visualize the distribution (Fig. 5).

## III. Supplementary Note

**Fitting fluorescence intensity versus particle radial distance.** We generated a customized fit to evaluate NarK-GFP and NirS-dsRed fluorescence intensities as functions of radial distance within particles. We assumed that gene expression intensity was controlled by two factors, oxygen ($O_2$) and nitrate ($NO_3^-$) concentration. $O_2$ inhibits expression whereas $NO_3^-$ should have a direct proportional relationship. These concentrations further naturally change with distance across the particle and with time.

Following previous results[7], we assumed an inhibitory relationship with $O_2$ as an exponential decay of the form:

$$Inhib_{O_2} = A \times e^{-\gamma_{O_2}[O_2]}$$

$O_2$ concentrations themselves were assumed to be represented with distance, *x*, by a logistic function of the form:

$$[O_2] = 2[O_2]_{bulk}\left(1 - \frac{1}{1 + e^{-\gamma x}}\right)$$

$NO_3^-$ was assumed to have a directly proportional relationship with expression:

$$Stim_{NO_3^-} = C \times [NO_3^-]$$

$NO_3^-$ concentrations were also assumed to be well represented with distance, *x*, by a logistic function, where *E* is a parameter offsetting the rapid depletion of nitrate:

$$[NO_3^-] = [NO_3^-]_{bulk}\left(1 - \frac{1}{1 + e^{-\delta(x-\varepsilon)}}\right)$$

The combined effect on fluorescence intensity is therefore the product of oxygen inhibition and nitrate stimulation:

$$E = \alpha \times e^{-\beta\left[2\,[O_2]_{bulk}\left(1-\frac{1}{1+e^{-\gamma x}}\right)\right]} \times [NO_3^-]_{bulk}\left(1 - \frac{1}{1 + e^{-\delta(x-\varepsilon)}}\right)$$

whereby α, β, γ, δ, and ε are fitting parameters and *x* is the distance to the particle edge. This functional form was fit to all NarK-GFP and NirS-dsRed datasets. For all timepoints, values near the particle edge ($r < 100$ µm) were assumed as background and removed for fitting, except for one timepoint ($t = 40$ h) wherein values at the particle center ($r > 1200$ µm) were removed. Plots comparing fits to data subtracted the background specific to each particle, *e.g.* the mean value for all points $r < 100$ µm (rfu = 350) was subtracted from all values for NarK-GFP for $t = 18$ h (Supplementary Fig. 5a).

## IV. Supplementary References


1 Schobert, M. & Jahn, D. Anaerobic physiology of Pseudomonas aeruginosa in the cystic fibrosis lung. *International Journal of Medical Microbiology* **300**, 549-556, doi:10.1016/j.ijmm.2010.08.007 (2010).

2 Line, L. *et al.* Physiological levels of nitrate support anoxic growth by denitrification of Pseudomonas aeruginosa at growth rates reported in cystic fibrosis lungs and sputum. *Frontiers in Microbiology* **5**, doi:10.3389/fmicb.2014.00554 (2014).

3 Zhang, C., Xing, X. H. & Lou, K. Rapid detection of a gfp-marked Enterobacter aerogenes under anaerobic conditions by aerobic fluorescence recovery. *Fems Microbiology Letters* **249**, 211-218, doi:10.1016/j.femsle.2005.05.051 (2005).

4 Menolascina, F. *et al.* Logarithmic sensing in Bacillus subtilis aerotaxis. *npj Systems Biology and Applications* **3**, doi:10.1038/npjsba.2016.36 (2017).

5 Klawonn, I., Bonaglia, S., Bruchert, V. & Ploug, H. Aerobic and anaerobic nitrogen transformation processes in N-2-fixing cyanobacterial aggregates. *Isme Journal* **9**, 1456-1466, doi:10.1038/ismej.2014.232 (2015).

6 Koren, K., Jakobsen, S. L. & Kuhl, M. In-vivo imaging of O-2 dynamics on coral surfaces spray-painted with sensor nanoparticles. *Sensors and Actuators B-Chemical* **237**, 1095-1101, doi:10.1016/j.snb.2016.05.147 (2016).

7 Dalsgaard, T. *et al.* Oxygen at Nanomolar Levels Reversibly Suppresses Process Rates and Gene Expression in Anammox and Denitrification in the Oxygen Minimum Zone off Northern Chile. *Mbio* **5**, doi:10.1128/mBio.01966-14 (2014).
