# Supplementary material for: Denitrifying bacteria respond to and shape microscale gradients within particulate matrices
Source: Commun Biol. 2021 May 13;4:570. doi: 10.1038/s42003-021-02102-4 (PMC8119678; doi:10.1038/s42003-021-02102-4)
Supplement: Supplementary file 3 — Description of Additional Supplementary Files [file 42003_2021_2102_MOESM3_ESM.pdf]

Description of Additional Supplementary Files

File Name: Supplementary Video 1.
Description: Microcolony growth in a particle over 24 h. *Pseudomonas aeruginosa* PAO1 (both NarK-GFP and NirS-dsRed strains, in equal portions) were embedded in an agarose particle disc (3 mm diameter) and incubated in a gastight millifluidic device. LB media supplemented with NO_3^- (1 mM) continually flowed past the particle at a velocity of ~0.7 mm s^{-1}. Images were acquired every 30 min. Scale bar = 750 µm.

File Name: Supplementary Data S1
Description: The data underlying Fig. 1.

File Name: Supplementary Data S2
Description: The data underlying Figs. 3 and 4 and Supplementary Figs. 5–8.

File Name: Supplementary Data S3
Description: The data underlying Fig. 2 and Supplementary Fig. 4.

File Name: Supplementary Data S4
Description: The data underlying Fig. 5.
